# An Aging-Related Gene Signature-Based Model for Risk Stratification and Prognosis Prediction in Lung Adenocarcinoma

**DOI:** 10.3389/fcell.2021.685379

**Published:** 2021-07-02

**Authors:** Qian Xu, Yurong Chen

**Affiliations:** ^1^Health Management Center, First Affiliated Hospital of Nanchang University, Nanchang, China; ^2^Department of Medical Oncology, Zhuji People’s Hospital of Zhejiang Province, Zhuji Affiliated Hospital of Shaoxing University, Zhuji, China

**Keywords:** lung adenocarcinoma, gene expression signature, aging, risk stratification analysis, biomarker

## Abstract

Aging is an inevitable time-dependent process associated with a gradual decline in many physiological functions. Importantly, some studies have supported that aging may be involved in the development of lung adenocarcinoma (LUAD). However, no studies have described an aging-related gene (ARG)-based prognosis signature for LUAD. Accordingly, in this study, we analyzed ARG expression data from The Cancer Genome Atlas (TCGA) and Gene Expression Omnibus (GEO). After LASSO and Cox regression analyses, a six ARG-based signature (*APOC3*, *EPOR*, *H2AFX*, *MXD1*, *PLCG2*, and *YWHAZ*) was constructed using TCGA dataset that significantly stratified cases into high- and low-risk groups in terms of overall survival (OS). Cox regression analysis indicated that the ARG signature was an independent prognostic factor in LUAD. A nomogram based on the ARG signature and clinicopathological factors was developed in TCGA cohort and validated in the GEO dataset. Moreover, to visualize the prediction results, we established a web-based calculator yurong.shinyapps.io/ARGs_LUAD/. Calibration plots showed good consistency between the prediction of the nomogram and actual observations. Receiver operating characteristic curve and decision curve analyses indicated that the ARG nomogram had better OS prediction and clinical net benefit than the staging system. Taken together, these results established a genetic signature for LUAD based on ARGs, which may promote individualized treatment and provide promising novel molecular markers for immunotherapy.

## Introduction

Lung cancer (LC) is the most commonly diagnosed malignancy worldwide and the leading cause of cancer-related mortality (Siegel et al., 2020). Histologically, the most common type of LC is non-small cell LC (NSCLC), which accounts for approximately 90% of LC cases (Goldstraw et al., 2011). There are three major types of NSCLC, i.e., lung adenocarcinoma (LUAD), squamous cell carcinoma, and large-cell cancer (Santarpia et al., 2018). Despite advances in diagnosis and treatment, the 5-year survival rate remains low, and the prognosis of most patients is poor (Wang J. et al., 2019), potentially because of the high heterogeneity of LUAD and the lack of effective diagnostic and prognostic biomarkers (Zeng et al., 2020). Therefore, it is still necessary to explore optimal therapeutic strategies and identify novel accurate biomarkers for assessing risk in patients with LUAD.

Aging is an inevitable time-dependent process associated with a gradual decline in many physiological functions and is also a major risk factor for cardiovascular, neurodegenerative, and neoplastic diseases (Otsuka et al., 2019). Cellular senescence is defined as irreversible growth arrest, leading to suppression of the uncontrolled proliferation of tumor cells (Campisi, 2005). Senescent cells exhibit an enlarged morphology and reduced motility compared with young cells; this may contribute to the suppression of cell migration, invasion, and metastasis (Chen et al., 2000). However, the effects of senescent cells on cancer are extremely complex. Aging-related genes (ARGs) play important roles in the regulation of cellular senescence and may therefore also affect tumor cells. Although ARGs inhibit tumors by regulating tumor cell senescence, they can also promote the development, invasion, metastasis, and progression of cancer (Henriksson and Lüscher, 1996; Ko and Prives, 1996; Johnson et al., 2013; Lee and Schmitt, 2019). Recently, the use of ARGs as diagnostic or prognostic molecular biomarkers has attracted the attention of researchers in the field of oncology (Yue et al., 2021). However, the prognostic roles of ARGs and their biological functions in LUAD remain unclear. In addition, no accurate ARG signature has been established for the prediction of LUAD-associated survival.

In general, an integrated model consisting of multiple genes will have greater predictive ability than a model including only a single gene (Srivastava and Gopal-Srivastava, 2002). Therefore, in this study, we aimed to develop a multiple ARG signature for prediction of LUAD clinical outcomes based on The Cancer Genome Atlas (TCGA) database. The performance of the signature was validated in a Gene Expression Omnibus (GEO) cohort. Finally, we also established a risk prognosis model based on the ARG signature to offer a more accurate prediction of LUAD prognosis than simple clinicopathologic nomograms.

## Materials and Methods

### Data Acquisition and Preparation

Data from the transcriptome analysis and related clinical information from patients with LUAD were obtained from TCGA^1^ and GEO databases^2^. After removing four cases with a follow-up of less than 1 day, 716 cases (490 cases from TCGA and 226 cases from GSE31210) with tumor samples and clinical data were ultimately included in analysis. TCGA data acted as a training set, whereas GSE31210 data acted as a validation set.

We obtained 307 human ARGs from the Human Aging Genomic Resources^3^. The ARGs are showed in Supplementary Table 1. The cBio Cancer Genomics Portal database (cBioPortal^4^) was used to evaluate mutations and copy number variations in tumor tissues.

### mRNA and Protein Levels Between Normal and Tumor Tissues

The Human Protein Atlas database^5^ was used to identify the protein expression of immunohistochemical staining of ARGs in LUAD patients. ARGs mRNA expression data of LUAD cohort were downloaded from TCGA-LUAD dataset (see text footnote 2).

### Construction and Validation of the Prognostic ARG Signature

To identify prognosis-related ARGs, we first performed univariate Cox analyses. The overlapping prognostic ARGs in TCGA and GEO was selected for subsequent studies. To further narrow down the ARGs, we performed least absolute shrinkage and selection operator (LASSO) regression in the training set. Finally, multivariate Cox regression analyses were then performed to establish a signature for predicting the survival of patients with LUAD in the training set using the “glmnet” R package. The formula of the risk score for the prediction of prognosis in patients with LUAD was as follows:

$$R\,i\,s\,k\,s\,c\,o\,r\,e=\sum_{\text{i}=0}^{\text{N}}\left(\beta \,\text{i}\times E\,\text{xp}\,i\right),$$

where N is the number of prognostic ARGs, Exp_i_ is the corresponding expression data for the identified ARGs, and β_i_ is the regression coefficient derived from the LASSO Cox regression model coefficients. LUAD cases were divided into high- and low-risk groups according to the median risk score. Kaplan–Meier survival analysis was performed to evaluate the association of overall survival (OS) and relapse-free survival (RFS) with the ARG signature.

### Functional Enrichment Analysis

The potential mechanisms of the ARG signature were explored using Gene Set Enrichment Analysis (GSEA) annotations. To explore different molecular mechanisms and pathways between high- and low-risk patients, Gene Ontology (GO) and Kyoto Encyclopedia of Genes and Genomes (KEGG) analyses were conducted using the R package. Genes with a false discovery rate of less than 0.05 after performing 1,000 permutations were considered significantly enriched.

### Immune Infiltration Analysis

Fractions of 22 human immune cell subsets in LUAD samples were calculated with Cell-type Identification by Estimating Relative Subsets of RNA Transcripts^6^, which is a computational program for characterizing immune cell compositions based on gene expression profiles (Tang et al., 2020). The proportion of each infiltrating immune cell was calculated based on the absolute mode between the low- and high-risk groups.

### Development of a Nomogram Based on ARGs and Clinical Factors

To construct a scoring system capable of evaluating the 1-, 3-, and 5-year OS of the patients, we established a nomogram in TCGA training cohort based on the ARG signature and other clinical factors. Calibration plots and area under the curve (AUC) in receiver operating characteristic (ROC) curves were applied to assess the predictive accuracy. In addition, decision curve analysis (DCA) was carried out to assess the clinical utility of the model. These tests were all performed both in the training set and validation set. Moreover, to facilitate clinical application, we established a visualization tool by a web-based calculator (Tang et al., 2021).

### Statistical Analysis

The expression profiles of the mRNAs are shown as raw data, and each mRNA was log_2_ normalized for further analysis. Continuous variables with normal distributions were presented as means ± standard deviations, and categorical variables were presented as percentages. Results with *p*-values less than 0.05 were used as a cutoff for statistical significance. Statistical analysis was performed using SPSS software version 24.0 and R 3.6.2 software.

## Results

### Identification of a Prognosis-Related ARG-Based Signature

**FIGURE 1:**
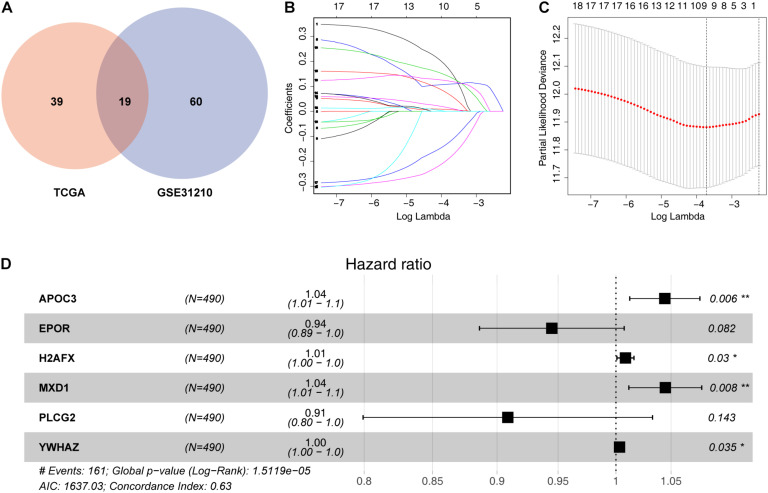
Identification of a prognosis-related ARG-based signature. **(A)** Nineteen overlapping prognostic ARGs based on TCGA and GSE31210. **(B)** Selection of the optimal parameter (lambda) in the least absolute shrinkage and selection operator (LASSO) model; dotted vertical lines were drawn at the optimal values using the minimum criteria. **(C)** LASSO coefficient profiles of the 19 prognosis-associated ARGs with non-zero coefficients determined by the optimal lambda. **(D)** Multivariate Cox regression analysis screened six ARGs (*APOC3*, *EPOR*, *H2AFX*, *MXD1*, *PLCG2*, and *YWHAZ*) to construct a risk signature. *P < 0.05 and **P < 0.01.

To identify survival-related ARGs, we first performed univariate Cox analyses using data for mRNA levels of each ARG. We identified 58 and 79 ARGs showing correlations with OS in patients with LUAD from TCGA and GEO databases, respectively (Supplementary Tables 2,3). Finally, 19 overlapping prognostic ARGs was screened for subsequent analysis (Figure 1A).

To further decrease the number of genes in the signature, the 19 ARGs were subjected to LASSO regression analysis (Figures 1B,C). Then, nine genes from LASSO were subjected to multivariate Cox regression analysis to develop a risk signature (Figure 1D). Finally, a risk signature including six ARGs was constructed based on 490 LUAD cases in the training set. The prognostic risk score formula was specifically constructed according to a linear combination of the expression levels weighted with the regression coefficients from the multivariate Cox regression analysis, as follows: risk score = *APOC3* × 0.043262193 – *EPOR* × 0.056851922 + *H2AFX* × 0.008373958 + *MXD1* × 0.043 730173 – *PLCG2* × 0.095876138 + *YWHAZ* × 0.003148576.

### mRNA and Protein Expression Profile and Alterations in LUAD

**FIGURE 2:**
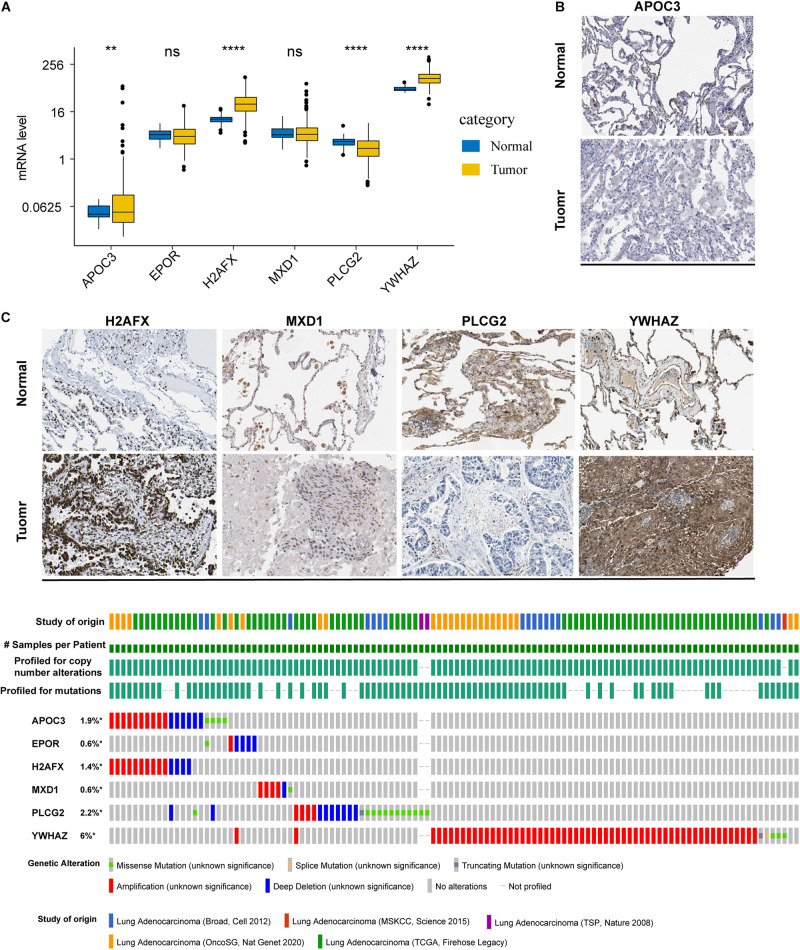
mRNA and protein expression profile and alterations of the ARGs in LUAD. **(A)** The mRNA expression profiles of ARGs between normal and tumor tissues in the training set. **P < 0.01 and ****P < 0.0001. **(B)** Representative immunohistochemical pictures of ARGs protein expression which were obtained from the Human Protein Atlas database (the data of EPOR were not available). **(C)** Genetic alterations in the ARGs in LUAD were determined using the cBioPortal database. *P < 0.05.

To determine the mRNA and protein levels of the six ARGs, we analyzed the RNA-seq data of the ARGs between normal and tumor tissues in the training set. We found that the mRNA levels of *APOC3*, *H2AFX*, and *YWHAZ* were significantly increased in patients, whereas *PLCG2* mRNA levels were significantly decreased (Figure 2A). However, relative mRNA expression levels of *EPOR* and *MXD1* were not significantly different between normal and tumor cases. By analyzing the ARGs protein expression profiles in HPA, we found that protein levels of the ARGs were approximately consistent with their mRNA expression levels (Supplementary Figure 1). Representative immunohistochemical pictures of ARGs protein expression were shown in Figure 2B.

The cBioPortal database was accessed to analyze the six ARGs of five different LUAD datasets. The results showed that the frequencies of gene alterations, including amplifications, deep deletions, and missense mutations, ranged from 0.6 to 6% (Figure 2C).

### GSEA of Risk-Dependent Groups

**FIGURE 3:**
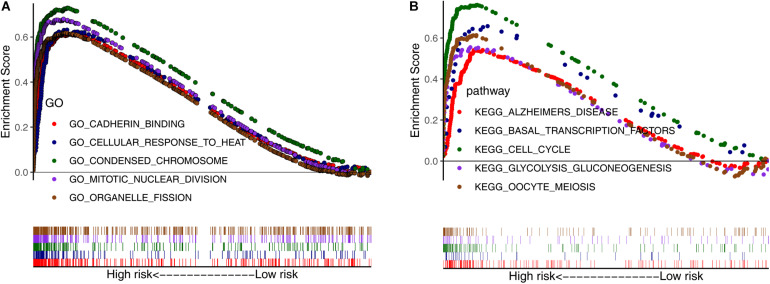
Gene set enrichment analysis between the low- and high-risk subgroups. **(A)** Enriched GO terms between high- and low-risk groups. **(B)** Enriched KEGG pathways between high- and low-risk groups.

Next, we conducted GSEA using GO and KEGG pathway enrichment analyses to further investigate the potential functional mechanisms leading to differential prognoses for patients with LUAD in the low- and high-risk subgroups in TCGA cohort. The ARGs were primarily enriched in oocyte meiosis, glycolysis gluconeogenesis, cell cycle, basal transcription factors, organelle fission, mitotic nuclear division, condensed chromosome, and cellular response to heat (Figures 3A,B). These results may provide insights into the cellular biological effects of the ARGs.

### Prognostic Value of the ARG Signature in the Training Set

**FIGURE 4:**
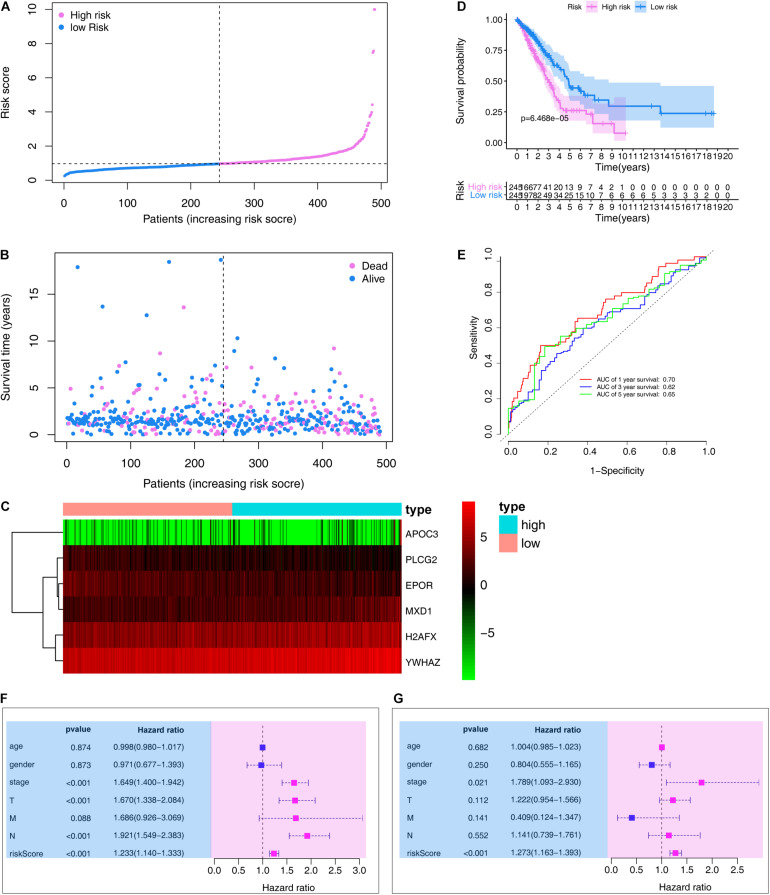
Evaluation the prognostic value of the ARG signature in the training set. **(A)** Distribution of the risk scores calculated by the risk score. **(B)** Patient distribution in the low- and high-risk score groups based on survival status. **(C)** Heatmap of the ARG expression profiles. **(D)** Overall survival curves stratified by the low- and high-risk group. **(E)** Time-dependent ROC curves for ARG-based overall survival prediction. **(F)** Univariate Cox regression analysis of signature and other clinical factors. **(G)** Multivariate Cox regression analysis of signature and other clinical factors.

We then ranked risk scores from low to high and divided sample data into low- and high-risk groups according to median value (Figure 4A). LUAD vital status and follow-up time for each individual are shown in Figure 4B. Additionally, a heatmap showing the expression profiles of the six ARGs was plotted (Figure 4C). Kaplan–Meier survival curves of low- and high-risk groups in the training set are shown in Figure 4D (*P* < 0.001).

In the training set, the signature was evaluated using time-dependent ROC curve analysis within different years. The AUC values of the 1-, 3-, and 5-year OS probability in the training set were 0.70, 0.62, and 0.65, respectively (Figure 4E). Moreover, we also performed Cox regression analysis, which showed that the ARG signature was an independent predictor following adjustment of clinicopathological features (Figures 4F,G).

**FIGURE 5:**
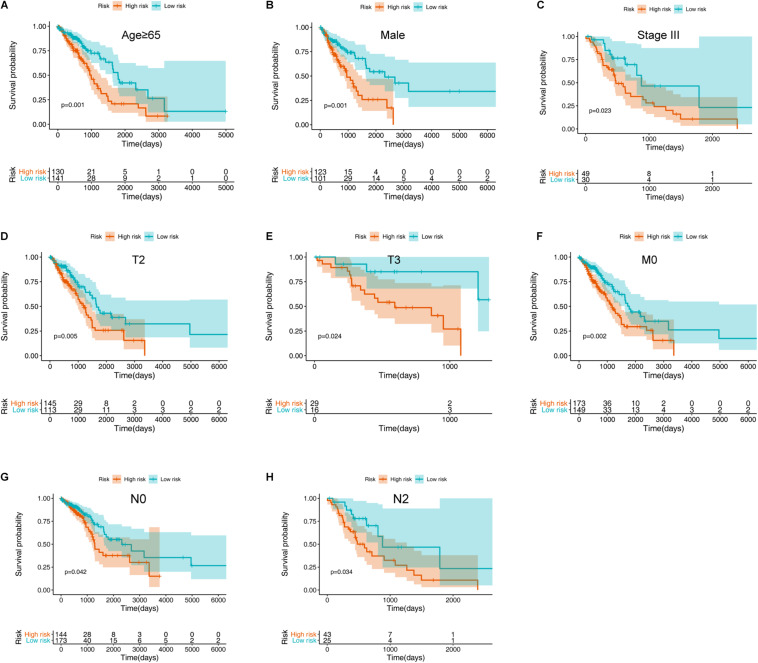
Assessment of the ARG signature via stratification of patients based on specific demographic and clinical features in the training set. **(A)** Age ≥ 65 years. **(B)** Male sex. **(C)** Stage III disease. **(D)** T2 disease. **(E)** T3 disease. **(F)** M0 disease. **(G)** N0 disease. **(H)** N2 disease.

To further validate the prognostic value of the ARG signature for various demographic and clinical characteristics, we performed subgroup analysis of data from TCGA training set. The association remained markedly significant in the multivariate Cox model after controlling for age, sex, and clinical stage (Figure 5 and Table 1).

**TABLE 1:** Prognostic roles of the ARGs signature with different demographic and clinical characteristics in TCGA training set (OS).

Characteristics	Number (high-/low-risk group)	%	HR (95% CI)	*P*-value
**Age (years)**				
≥65	130/141	55.3%	2.062 (1.357–3.132)	0.001
<65	115/104	44.7%	1.636 (0.995–2.690)	0.052
**Sex**				
Female	122/144	54.3%	1.516 (0.978–2.349)	0.063
Male	123/101	45.7%	2.347 (1.435–3.839)	0.001
**Stage**				
I	109/152	53.3%	1.329 (0.772–2.288)	0.306
II	69/48	23.9%	1.844 (0.994–3.422)	0.052
III	49/30	16.1%	2.290 (1.122–4.671)	0.023
IV	14/11	5.1%	1.450 (0.482–4.355)	0.508
NA	4/4	1.6%	−	−
**T stage**				
T1	59/107	33.9%	1.420 (0.751–2.686)	0.280
T2	145/113	52.7%	1.866 (1.206–2.889)	0.005
T3	29/16	9.2%	5.638 (1.261–25.205)	0.024
T4	11/7	3.7%	1.274 (0.316–5.138)	0.733
NA	1/2	0.6%	−	−
**M stage**				
M0	173/149	65.7%	1.847 (1.241–2.747)	0.002
M1	14/10	4.9%	1.699 (0.520–5.549)	0.380
NA	58/86	29.4%	−	−
**N stage**				
N0	144/173	64.7%	1.622 (1.01902.583)	0.042
N1	53/39	18.8%	1.523 (0.834–2.781)	0.171
N2	43/25	13.9%	2.264 (1.063–4.825)	0.034
N3	1/1	0.4%	−	−
NA	4/7	2.2%	−	−

**NA, not available; HR, hazard ratio; CI, confidence interval; OS, overall survival.**

### Prognostic Value of the ARG Signature in the Validation Set

**FIGURE 6:**
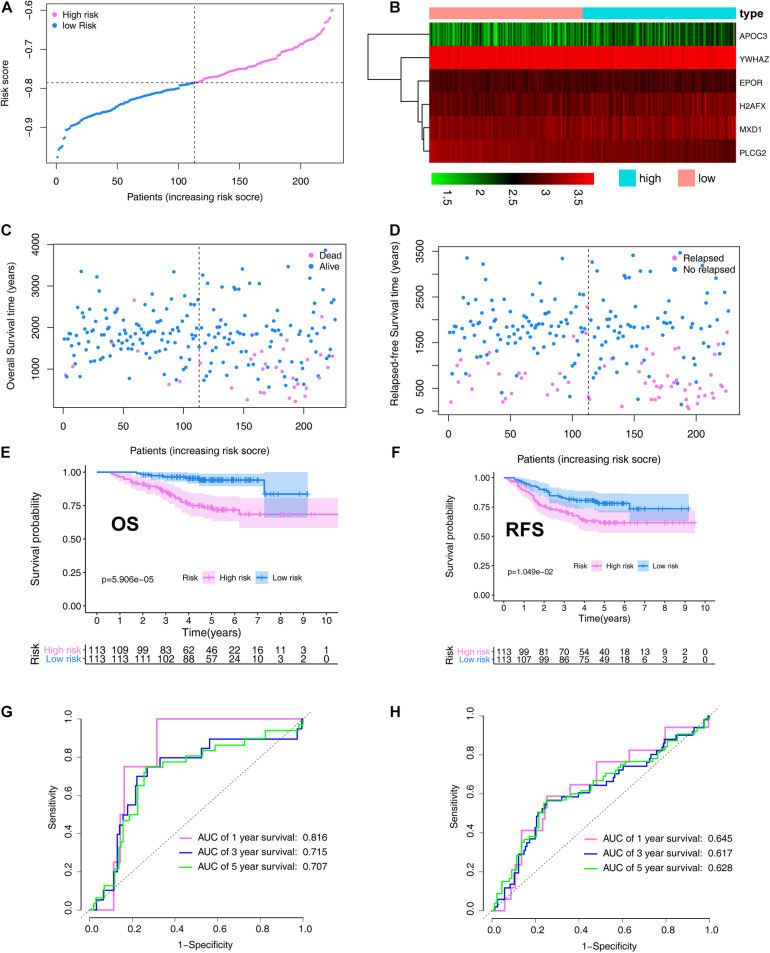
Verification the prognostic value of the ARG signature in the validation set (GSE31210). **(A)** Distribution of the risk scores calculated by the risk score. **(B)** Heatmap of the ARG expression profiles based on the RNA-seq data. **(C)** Patient distribution in the low- and high-risk score groups based on overall survival status. **(D)** Patient distribution in the low- and high-risk score groups based on relapse-free survival status. **(E)** Overall survival curves stratified by the low- and high-risk group. **(F)** Relapse-free survival curves stratified by the low- and high-risk group. **(G)** Time-dependent ROC curves for ARG-based overall survival prediction. **(H)** Time-dependent ROC curves for ARG-based relapse-free survival prediction.

In the validation set, patients with LUAD were divided into high- (*N* = 113) and low-risk (*N* = 113) cohorts based on the median risk score. The risk score distribution is shown in Figure 6A. A heatmap showing the expression profiles of the six ARGs was plotted (Figure 6B). The LUAD OS status and follow-up time for each individual are shown in Figure 6C. LUAD RFS status and follow-up time for each individual are shown in Figure 6D. OS and RFS curves for low- and high-risk groups in the training set are shown in Figures 6E,F, respectively.

The signature was evaluated using time-dependent ROC curve analysis within different years. The AUC values of the 1-, 3-, and 5-year OS probability in the validation set were 0.816, 0.715, and 0.707, respectively (Figure 6G). The AUC values of the 1-, 3-, and 5-year RFS probability in the validation set were 0.645, 0.617, and 0.628, respectively (Figure 6H).

To further validate the prognostic value of the ARG signature based on various demographic and clinical characteristics, we performed subgroup analysis of the GEO validation set. The association remained markedly significant in the multivariate Cox model after controlling for age, sex, and clinical stage (Table 2).

**TABLE 2:** Prognostic roles of the ARGs signature with different demographic and clinical characteristics in GSE31210 validation set.

Characteristics	Number (high-/low-risk group)	%	OS		RFS	
			HR (95% CI)	*P*-value	HR (95% CI)	*P*-value
**Age (years)**						
≥65	29/33	27.4%	4.847 (1.348–17.425)	0.16	3.162 (1.287–7.766)	0.012
<65	84/80	72.6%	4.780 (1.606–14.222)	0.005	1.530 (0.826–2.834)	0.177
**Sex**						
Female	59/62	53.5%	8.550 (1.941–37.668)	0.005	1.721 (0.850–3.487)	0.132
Male	54/51	46.5%	3.200 (1.151–8.898)	0.026	2.144 (1.033–4.449)	0.041
**Smoking status**						
Ever smoker	58/53	49.1%	3.348 (1.215–9.228)	0.019	2.259 (1.101–4.636)	0.026
Never smoker	55/60	50.9%	7.926 (1.786–35.175)	0.006	1.586 (0.770–3.269)	0.211
**Stage**						
I	78/90	74.3%	4.085 (1.331–12.533)	0.014	1.595 (0.832–3.057)	0.160
II	35/23	25.7%	4.817 (1.385–16.747)	0.013	2.152 (0.939–4.933)	0.070
**Mutation**						
*ALK* fusion	10/1	4.9%	23.412(0.000–2.365E + 10)	0.766	23.670 (0.000–1.402E + 10	0.759
*EGFR* mutation	58/69	56.2%	4.833 (1.347–17.348)	0.016	1.717 (0.833–3.537)	0.143
*KRAS* mutation	12/8	8.8%	48.345 (0.002–973572.971)	0.443	57.911 (0.078–43074.053)	0.229
Wild-type *EGFR/KRAS/ALK*	33/35	30.1%	3.901 (1.253–12.147)	0.019	1.705 (0.782–3.714)	0.180

**HR, hazard ratio; CI, confidence interval; OS, overall survival; RFS, relapsed-free survival; ALK, anaplastic lymphoma kinase; KRAS, Kirsten rat sarcoma viral oncogene homolog; EGFR, epidermal growth factor receptor.**

### ARGs and Correlations With Clinicopathological Characteristics

**FIGURE 7:**
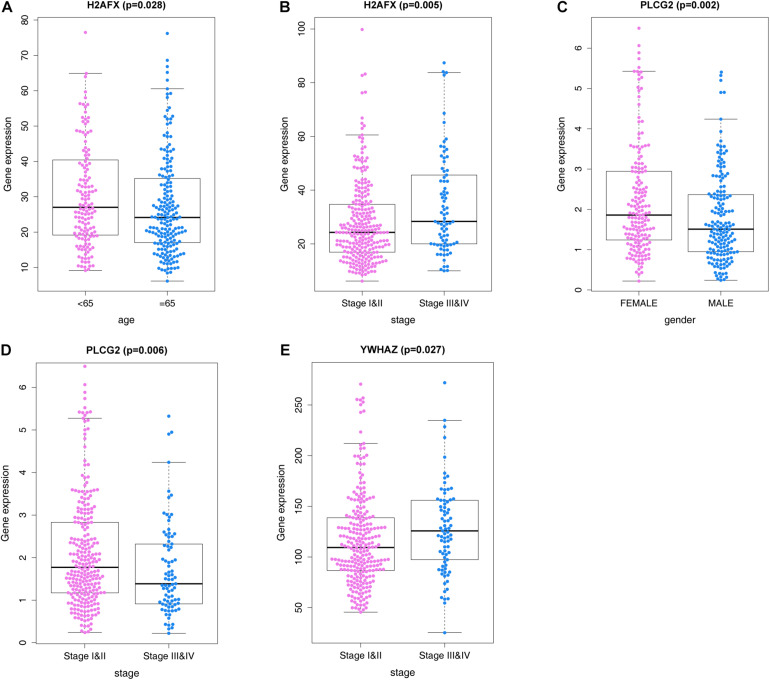
ARGs and correlations with clinicopathological characteristics. **(A)** *H2AFX* and age. **(B)** *H2AFX* and stage. **(C)** *PLCG2* and sex. **(D)** *PLCG2*, and stage. **(E)** *YWHAZ* and stage.

We analyzed correlations of the ARG signature with clinicopathological features, including age, sex, and pathological tumor/node/metastasis (TNM) stage, in the training set, and explored the relationships between the screened ARGs and clinical indices. The results suggested that *H2AFX*, *PLCG2*, and *YWHAZ* were differentially expressed in patients with various clinical features (Figure 7).

### Analysis of Tumor Immunity

**FIGURE 8:**
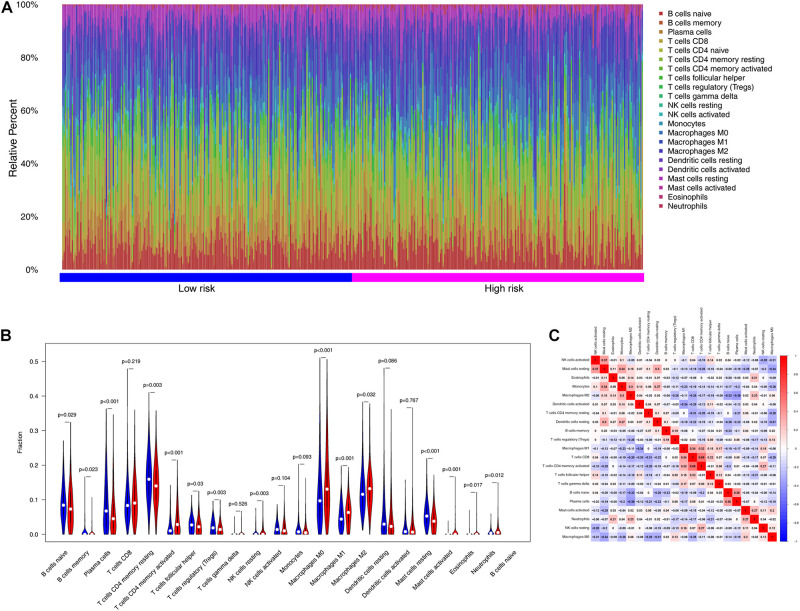
The landscape of immune infiltration between high- and low-risk groups in the training set. **(A)** Immune cell type percentages in the low- and high-risk groups. **(B)** Differential immune infiltrates in the high-risk and low-risk groups. **(C)** Correlation matrix of the relationship between the expression levels of the six ARGs and differential immune infiltration levels.

To examine the relationships between risk scores and tumor immunity, we utilized the ESTIMATE algorithm to process TCGA data. Figure 8A shows the immune cell type percentages in the low- and high-risk groups from the training dataset. When we conducted an immune cell type-specific analysis, we found that low-risk patients exhibited higher levels of naïve B cells, memory B cells, plasma cells, resting CD4 memory T cells, follicular helper T cells, regulatory T cells (Tregs), and resting mast cells. High-risk patients exhibited higher levels of activated CD4 memory T cells, activated natural killer (NK) cells, M0 macrophages, M1 macrophages, M2 macrophages, activated mast cells, eosinophils, and neutrophils (Figure 8B). Figure 8C also demonstrates correlations between the immune cell types.

### Development of an ARG Nomogram to Predict Individual Outcomes of LUAD

**FIGURE 9:**
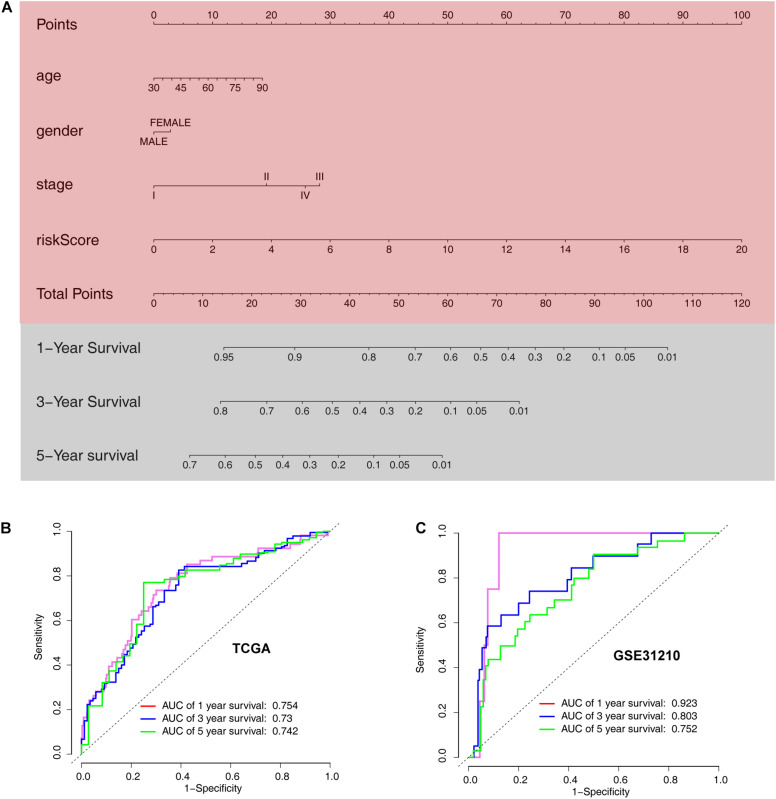
Development of a nomogram model based on clinical characteristics and the 6-ARG signature. **(A)** Nomogram model combined with stage, age, and ARG signature. **(B)** Receiver operating characteristic curves for model-based overall survival prediction. **(C)** Receiver operating characteristic curves for model-based relapse-free survival prediction.

The ARG signature and other three valuable factors (age, stage, and sex) were selected to establish a predictive nomogram, which is an intuitive visualization of the model to predict survival probability at 1-, 3-, and 5- years, based on data from TCGA training set (Figure 9A). The nomogram was evaluated using time-dependent ROC curve analysis within 1, 3, and 5 years. The AUC values of the 1-, 3-, and 5-year OS probability in the training set were 0.754, 0.73, and 0.42, respectively (Figure 9B). The AUC values of the 1-, 3-, and 5-year OS probability in the training set were 0.923, 0.752, and 0.42, respectively (Figure 9C).

**FIGURE 10:**
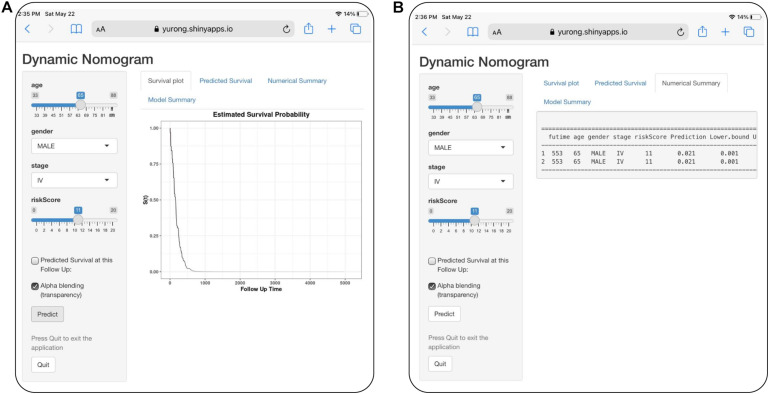
Development of an easy-to-operate web-based calculator for predicting overall survival in patients with LUAD (https://jiangyanxia.shinyapps.io/LUAD/). **(A)** Web-based overall survival rate calculator. **(B)** The 95% confidence interval of the web-based progression-free survival rate.

For visualization and convenient clinical use of the prognostic nomogram, we developed an easy-to-operate web-based model^7^ to predict the OS of LUAD based on the established nomogram (Figure 10). The estimated disease progression probabilities could be obtained by drawing a perpendicular line from the total point axis to the outcome axis.

**FIGURE 11:**
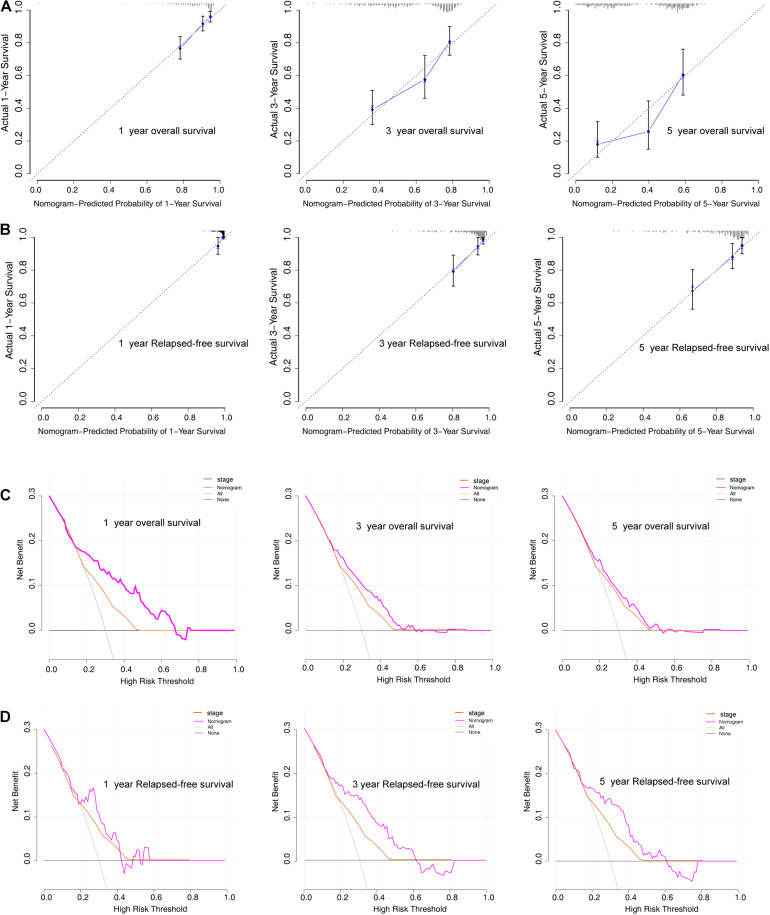
Model discrimination and performance in the training set. **(A,B)** Calibration plot examining predictive accuracy for 1-, 3-, and 5-year overall survival and relapse-free survival rate. **(C,D)** Decision curve analysis of the nomogram for 1-, 3-, and 5-year overall survival and relapse-free survival rate in LUAD patients.

To further evaluate the predictive performance and clinical usefulness of the prognostic nomogram, calibration curves and decision curve analysis (DCA) were performed. The calibration curves of the nomogram showed good probability consistencies between the prediction and observation in both the training and validation sets (Figures 11A,B). Furthermore, DCA confirmed our expectations, revealing that the net benefit in predictions was the highest in the combined nomogram model compared with the TNM staging system (Figures 11C,D).

## Discussion

Traditional clinical analyses, such as American Joint Committee on Cancer staging and assessment of pathological parameters, do not accurately or dynamically reflect LUAD progression and show poor prognostic capacity. Therefore, adequate and accurate assessments and prediction tools are essential for treatment decision-making and prognostic evaluation in patients with LUAD. Currently, the role of the aging process in LUAD is still unclear. Exploring the expression patterns of ARGs is crucial for understanding the role of the aging process in LUAD. Therefore, in this study, we assessed the involvement of ARGs in prognosis in patients with LUAD.

In the current study, we developed an aging-based signature of six ARGs, including *APOC3*, *EPOR*, *H2AFX*, *MXD1*, *PLCG2*, and *YWHAZ*. The aging signature was identified as an independent risk factor for patients with LUAD and was significantly associated with prognosis in most clinical and mutation subgroups. Furthermore, an ARG-signature based nomogram and corresponding web-based calculator were established as references for clinicians to facilitate clinical decision-making.

Previous studies have shown attempts to establish models for predicting prognosis in patients with LUAD based on sequencing data and clinical features (Wang et al., 2020; Huang et al., 2021; Wang J. et al., 2021; Wang X. et al., 2021; Zhou et al., 2021). However, limited results have been applied in clinical practice. In the current study, our nomogram showed good calibration and discrimination, with AUC values generated to predict 1-, 3-, and 5-year progression-free survival in the training set of 0.754, 0.73, and 0.742, respectively. In the validation set, the model still showed excellent calibration and discrimination, with AUC values for predicting 1-, 3-, and 5-year progression-free survival in the training set of 0.923, 0.803, and 0.752, respectively. These results showed that the approach had excellent predictive ability for survival in patients with LUAD. Moreover, we developed an easy-to-operate calculator that will enable the public to freely predict local cases and diagnose the adaptability of the model.

The majority of ARGs included in our signature are closely related to tumor initiation, proliferation, and metastasis. Apolipoprotein C3 (APOC3) is primarily present on the surface of triglyceride-rich lipoproteins and has been reported as an independent contributor to triglyceride levels (Marín-Vicente et al., 2020). APOC3 is a potential prognostic biomarker for several types of malignant tumors (Wang X. et al., 2019; Marín-Vicente et al., 2020). Erythropoietin receptor (EPOR), a glycoprotein produced in the fetal liver and adult kidney, is the chief regulator of erythropoiesis (Fisher, 2003; Oshima et al., 2021). Although EPOR has been shown to be expressed in NSCLC, its value as a potential prognostic marker in NSCLC is still unclear (Saintigny et al., 2007; Rózsás et al., 2013; Patterson et al., 2015). H2A histone family, member X (H2AFX) has been reported to be involved in the DNA repair pathway (Santarpia et al., 2013; Huang et al., 2014). Several studies have shown that H2AFX is dysregulated in lung cancer (Castro et al., 2010; Caramori et al., 2011; Corveloni et al., 2020). However, the specific mechanisms through which H2AFX affects tumor progression have not been elucidated. MAX dimerization protein 1 (MXD1) belongs to the MAX dimerization protein family of proteins, which function as potent antagonists of c-Myc (Thomas et al., 2015). MXD1 contributes to tumor initiation in various tissues (Biyajima et al., 2015). However, the roles of MXD1 in lung cancer remain largely unexplored. *PLCG2* encodes the phosphoinositide specific phospholipase C family protein PLCG2, which is critical for the modulation of calcium signals in response to immune receptor stimulation (Wilde and Watson, 2001). However, few reports have described the roles of PLCG2 in LUAD. Tyrosine 3-monooxygenase/tryptophan 5-monooxygenase activation protein zeta (YWHAZ) is involved in many vital cellular processes, such as metabolism, protein trafficking, signal transduction, apoptosis, and cell cycle regulation (Gong et al., 2021). Emerging evidence has shown that YWHAZ plays critical roles in the progression of many types of tumors, including lung cancer (Chen et al., 2012).

To improve our understanding of the mechanisms through which ARGs may mediate differential prognoses in patients with LUAD, we also performed GSEA for low- and high-risk groups based on our ARG signature. In this analysis, we identified oocyte meiosis, glycolysis gluconeogenesis, cell cycle, basal transcription factors, organelle fission, mitotic nuclear division, condensed chromosome, and cellular response to heat as major pathways involving ARGs. These results provide a reference for further studies on the mechanisms of ARGs in LUAD.

Cellular senescence often triggers an immune response in the tumor microenvironment (Song et al., 2020), and immune cell infiltration into the tumor microenvironment promotes tumor growth (Sarode et al., 2020). However, the modulation of immune cells by ARGs is relatively unclear in LUAD. In this study, the high-risk group, established based on the expression levels of ARGs, showed higher infiltration proportions of activated CD4 memory T cells, activated NK cells, M0 macrophages, M1 macrophages, M2 macrophages, activated mast cells, eosinophils, and neutrophils. However, low-risk patients exhibited higher levels of naïve B cells, memory B cells, plasma cells, resting CD4 memory T cells, follicular helper T cells, Tregs, and resting mast cells. Future studies, however, will be required to elucidate the exact prognostic relevance of these cells.

This study had some limitations. First, more independent LUAD cohorts should be used to validate the identified prognostic ARGs. Second, the results obtained only through bioinformatics analysis are inadequate, and experimental validation is needed to confirm these results. Therefore, further studies are needed to investigate the underlying mechanisms associated with the prognostic significance of the identified ARGs in LUAD.

In conclusion, we constructed and validated a model based on the ARG signature and other clinical features for predicting prognosis in patients with LUAD. This model could serve as a reliable tool for determining treatment strategies and potential outcomes in patients with LUAD.

## Data Availability Statement

The datasets presented in this study can be found in online repositories. The names of the repository/repositories and accession number(s) can be found in the article/Supplementary Material.

## Author Contributions

QX designed the study and performed the data analysis statistical analysis. YC prepared the manuscript. Both authors read and approved the final manuscript.

## Conflict of Interest

The authors declare that the research was conducted in the absence of any commercial or financial relationships that could be construed as a potential conflict of interest.

## Footnotes

1 https://tcga-data.nci.nih.gov/tcga/

2 https://www.ncbi.nlm.nih.gov/geo/

3 http://genomics.senescence.info/genes/

4 http://www.cbioportal.org/

5 https://www.proteinatlas.org/

6 https://cibersort.stanford.edu/

7 https://yurong.shinyapps.io/ARGs_LUAD/
