# Supplementary figures and images for: An Aging-Related Gene Signature-Based Model for Risk Stratification and Prognosis Prediction in Lung Adenocarcinoma
Source: Front Cell Dev Biol. 2021 Jul 2;9:685379. doi: 10.3389/fcell.2021.685379 (PMC8283194; doi:10.3389/fcell.2021.685379)

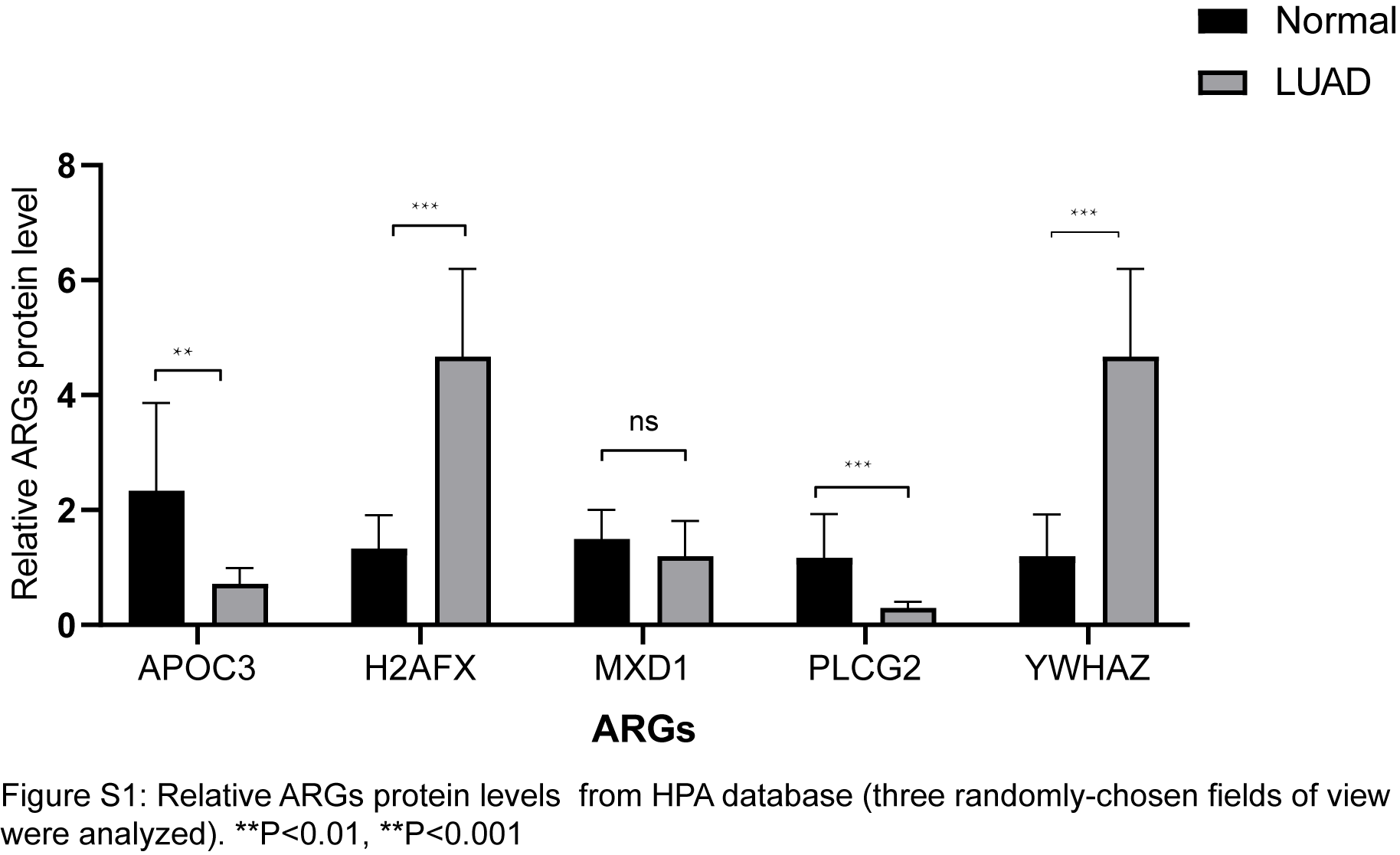

Supplement: Supplementary file 1 [file Image_1.TIF]
