# Supplementary material for: An Aging-Related Gene Signature-Based Model for Risk Stratification and Prognosis Prediction in Lung Adenocarcinoma
Source: Front Cell Dev Biol. 2021 Jul 2;9:685379. doi: 10.3389/fcell.2021.685379 (PMC8283194; doi:10.3389/fcell.2021.685379)
Supplement: Supplementary file 3 [file Table_2.DOCX]

| **Tabe S2. Prognosis-related ARGs inTCGA.** | | | | |
| --- | --- | --- | --- | --- |
| **Gene** | **HR** | **HR.95L** | **HR.95H** | **pvalue** |
| APOC3 | 1.040089683 | 1.007886269 | 1.073322042 | 0.01430613 |
| BAK1 | 1.030246052 | 1.009343269 | 1.051581717 | 0.004382867 |
| BDNF | 1.226073575 | 1.051389619 | 1.429780535 | 0.009350163 |
| BLM | 1.103854197 | 1.022799208 | 1.191332647 | 0.011107052 |
| BRCA2 | 1.274605097 | 1.01463935 | 1.601177949 | 0.03708383 |
| BUB1B | 1.050485312 | 1.014646518 | 1.087589985 | 0.005419809 |
| BUB3 | 1.054323006 | 1.021440904 | 1.088263645 | 0.001066952 |
| CAT | 0.987470461 | 0.977526411 | 0.997515669 | 0.014620384 |
| CCNA2 | 1.029304454 | 1.012915839 | 1.045958232 | 0.000420165 |
| CDK1 | 1.017358242 | 1.003253342 | 1.031661446 | 0.015694373 |
| CDKN1A | 1.006672731 | 1.000845162 | 1.012534233 | 0.024757792 |
| CEBPB | 1.005084174 | 1.000747472 | 1.00943967 | 0.021525369 |
| CSNK1E | 1.027122154 | 1.017144618 | 1.037197563 | 7.74E-08 |
| CTF1 | 0.963504473 | 0.928788965 | 0.999517548 | 0.047062352 |
| DBN1 | 1.01116546 | 1.000046579 | 1.022407966 | 0.049042694 |
| EGFR | 1.005200228 | 1.000393919 | 1.010029628 | 0.033920342 |
| EPOR | 0.940368699 | 0.884900299 | 0.999314037 | 0.047469669 |
| ERCC1 | 1.028245683 | 1.003684015 | 1.053408413 | 0.023941309 |
| FEN1 | 1.024095693 | 1.008563738 | 1.039866841 | 0.002261405 |
| FOXM1 | 1.029594035 | 1.013354082 | 1.04609425 | 0.000323996 |
| GCLC | 1.005130752 | 1.000170528 | 1.010115576 | 0.042609432 |
| GHRHR | 2.392536853 | 1.128733411 | 5.07137694 | 0.022852429 |
| GSK3A | 1.038722593 | 1.015616704 | 1.062354154 | 0.000932711 |
| H2AFX | 1.010576626 | 1.003656283 | 1.017544686 | 0.002691374 |
| HDAC2 | 1.060355717 | 1.017351293 | 1.10517798 | 0.005531598 |
| HSP90AA1 | 1.001679462 | 1.000348418 | 1.003012277 | 0.013381905 |
| HSPA1A | 1.004000907 | 1.000265124 | 1.007750642 | 0.035787088 |
| HSPA9 | 1.008156315 | 1.001721563 | 1.014632401 | 0.012901716 |
| HSPD1 | 1.005024039 | 1.002121096 | 1.007935391 | 0.000684669 |
| IGF1R | 1.03053825 | 1.004974806 | 1.056751948 | 0.01891636 |
| IGFBP3 | 1.00212827 | 1.000684442 | 1.003574181 | 0.003851597 |
| KCNA3 | 0.898889612 | 0.818143492 | 0.987604916 | 0.026440053 |
| LMNB1 | 1.017586416 | 1.006106995 | 1.029196814 | 0.002597142 |
| MXD1 | 1.052960016 | 1.022064238 | 1.084789736 | 0.000683074 |
| MYC | 1.007949423 | 1.001358333 | 1.014583897 | 0.018006296 |
| NBN | 1.050716725 | 1.021440048 | 1.080832533 | 0.000600776 |
| NFKB2 | 1.020463731 | 1.003495176 | 1.037719214 | 0.017894643 |
| NGF | 0.932291757 | 0.880320368 | 0.987331375 | 0.016592693 |
| NUDT1 | 1.036524288 | 1.002427591 | 1.071780755 | 0.035549762 |
| PARP1 | 1.013549325 | 1.002104113 | 1.025125255 | 0.020194048 |
| PIK3CA | 1.119750403 | 1.002098104 | 1.251215785 | 0.045828573 |
| PLCG2 | 0.870045299 | 0.76930912 | 0.983972246 | 0.026601226 |
| POU1F1 | 7.703113582 | 2.085123627 | 28.45776533 | 0.002198073 |
| PPARG | 1.027110489 | 1.001480141 | 1.053396781 | 0.038016382 |
| PRKCD | 0.970197434 | 0.953741581 | 0.986937216 | 0.000527372 |
| PRKDC | 1.018120897 | 1.006208383 | 1.030174444 | 0.002783882 |
| PTPN11 | 1.024860204 | 1.003755193 | 1.04640897 | 0.020721942 |
| RAD51 | 1.095149902 | 1.030775558 | 1.163544575 | 0.003275297 |
| SERPINE1 | 1.004340761 | 1.001817537 | 1.00687034 | 0.000738605 |
| SHC1 | 1.010447383 | 1.006377253 | 1.014533975 | 4.49E-07 |
| SOD1 | 1.003683538 | 1.000488164 | 1.006889117 | 0.023824781 |
| SOD2 | 1.009353572 | 1.002383699 | 1.016371909 | 0.008453445 |
| SP1 | 1.03580652 | 1.003279803 | 1.069387765 | 0.03068678 |
| TERT | 1.236484121 | 1.085834608 | 1.408034861 | 0.001363611 |
| TFDP1 | 1.001076482 | 1.000148217 | 1.002005608 | 0.023021247 |
| XRCC5 | 1.014444543 | 1.006909921 | 1.022035545 | 0.000163011 |
| XRCC6 | 1.005880527 | 1.001702352 | 1.010076129 | 0.005764671 |
| YWHAZ | 1.005165437 | 1.00256525 | 1.007772367 | 9.68E-05 |
