# Supplementary material for: An Aging-Related Gene Signature-Based Model for Risk Stratification and Prognosis Prediction in Lung Adenocarcinoma
Source: Front Cell Dev Biol. 2021 Jul 2;9:685379. doi: 10.3389/fcell.2021.685379 (PMC8283194; doi:10.3389/fcell.2021.685379)
Supplement: Supplementary file 4 [file Table_3.DOCX]

| **Tabe S3. Prognosis-related ARGs in GSE31210.** | | | | |
| --- | --- | --- | --- | --- |
| **id** | **HR** | **HR.95L** | **HR.95H** | **pvalue** |
| AGTR1 | 0.596250106 | 0.451849364 | 0.786798029 | 0.000257491 |
| APOC3 | 1.692922355 | 1.182803764 | 2.423044454 | 0.004006477 |
| APOE | 1.472013856 | 1.023347073 | 2.117389935 | 0.037125206 |
| BAX | 2.156122966 | 1.023338888 | 4.542841377 | 0.043317116 |
| BLM | 2.724144691 | 1.604969983 | 4.623740245 | 0.000205083 |
| BRCA1 | 1.892155313 | 1.248066574 | 2.868638424 | 0.002667203 |
| BRCA2 | 2.353584342 | 1.241656589 | 4.461265136 | 0.008707116 |
| BUB1B | 1.626534339 | 1.268885942 | 2.084989573 | 0.000123219 |
| CCNA2 | 1.630434958 | 1.128158493 | 2.356333945 | 0.009274851 |
| CDK1 | 1.674059503 | 1.241104232 | 2.258049848 | 0.000739004 |
| CISD2 | 2.626135284 | 1.06911767 | 6.450727289 | 0.035228325 |
| CLOCK | 0.168409948 | 0.051485675 | 0.550869943 | 0.003218312 |
| CREB1 | 0.072041279 | 0.013300779 | 0.390198643 | 0.002274939 |
| CREBBP | 0.213792772 | 0.073659841 | 0.620519246 | 0.004543705 |
| DDIT3 | 2.240965991 | 1.2536045 | 4.005991184 | 0.00647725 |
| EEF1E1 | 2.231008559 | 1.136763622 | 4.378570087 | 0.019670563 |
| EEF2 | 0.282961293 | 0.131400194 | 0.609337713 | 0.001256459 |
| EP300 | 0.324543031 | 0.142829036 | 0.737442345 | 0.007204125 |
| EPOR | 0.209975309 | 0.06928373 | 0.636363404 | 0.00579913 |
| ERCC3 | 0.18895305 | 0.059390074 | 0.601165357 | 0.004776365 |
| ERCC4 | 0.428582141 | 0.200668222 | 0.915354955 | 0.028640305 |
| FEN1 | 2.054571613 | 1.42084881 | 2.970945596 | 0.000129916 |
| FGF23 | 1.45747301 | 1.080989641 | 1.965076719 | 0.013483093 |
| FLT1 | 1.812895987 | 1.075975653 | 3.054522518 | 0.025412535 |
| FOXM1 | 1.654449843 | 1.290993219 | 2.120231341 | 6.95E-05 |
| GCLM | 1.762389425 | 1.142689896 | 2.718162205 | 0.010367336 |
| GHR | 0.685573874 | 0.490119006 | 0.958974313 | 0.027481544 |
| GRB2 | 6.191295724 | 2.140510652 | 17.90794299 | 0.000767186 |
| GRN | 2.544427692 | 1.211146795 | 5.3454398 | 0.013672594 |
| GSTP1 | 2.143527185 | 1.128511314 | 4.07147783 | 0.019842386 |
| H2AFX | 1.96292585 | 1.24117724 | 3.104373629 | 0.003928909 |
| HBP1 | 0.300153055 | 0.09426315 | 0.955748416 | 0.041694553 |
| HDAC2 | 2.610587294 | 1.203483443 | 5.662866459 | 0.015149995 |
| HELLS | 1.751388781 | 1.104079002 | 2.778209398 | 0.01728665 |
| HIF1A | 6.443564187 | 1.421270928 | 29.21295203 | 0.015700161 |
| HSF1 | 1.947576172 | 1.186739278 | 3.196197358 | 0.008355485 |
| HSPD1 | 4.629314093 | 1.351024321 | 15.86244499 | 0.014737037 |
| IFNB1 | 0.677778272 | 0.491702958 | 0.934270129 | 0.017541043 |
| IGFBP3 | 1.63979877 | 1.133322732 | 2.372616317 | 0.008691145 |
| IL7 | 0.648154425 | 0.421336184 | 0.997075908 | 0.048462526 |
| KL | 0.616797494 | 0.443462931 | 0.857882637 | 0.004097096 |
| LMNB1 | 1.746105105 | 1.115409924 | 2.733419324 | 0.014784113 |
| LRP2 | 0.767151169 | 0.604582493 | 0.973433605 | 0.029141658 |
| MAPK14 | 3.421188761 | 1.581426202 | 7.401251173 | 0.001783606 |
| MAPK3 | 3.212843414 | 1.141030195 | 9.046529046 | 0.027122761 |
| MAPT | 0.362035411 | 0.165329414 | 0.79277871 | 0.011065366 |
| MAX | 4.790150054 | 1.202992267 | 19.0737199 | 0.026275643 |
| MIF | 2.55592013 | 1.512157183 | 4.320138 | 0.000458024 |
| MXD1 | 1.570029526 | 1.024702581 | 2.405568952 | 0.038260423 |
| NCOR1 | 0.183919149 | 0.062712596 | 0.539385315 | 0.002038892 |
| NFE2L2 | 0.196460435 | 0.050001007 | 0.771918509 | 0.019766624 |
| NR3C1 | 0.238762849 | 0.107287557 | 0.531354239 | 0.000449429 |
| NUDT1 | 2.279356722 | 1.38886622 | 3.740797343 | 0.001115875 |
| PCNA | 2.54918411 | 1.402593194 | 4.633089377 | 0.002141681 |
| PIK3R1 | 0.465684039 | 0.268264074 | 0.808388618 | 0.006610203 |
| PLAU | 1.650728799 | 1.258672839 | 2.164903764 | 0.000291381 |
| PLCG2 | 0.587679021 | 0.357489093 | 0.966089985 | 0.036083053 |
| PML | 8.088831397 | 3.334278872 | 19.62319166 | 3.78E-06 |
| PON1 | 0.663686504 | 0.485757603 | 0.906789257 | 0.010040713 |
| PPARGC1A | 0.753682676 | 0.569330338 | 0.997729329 | 0.048172052 |
| PPM1D | 0.313964827 | 0.116247102 | 0.847968771 | 0.022296495 |
| RAD51 | 2.052826755 | 1.430489093 | 2.945913889 | 9.51E-05 |
| RECQL4 | 1.679421614 | 1.168275328 | 2.414205699 | 0.005111839 |
| RGN | 0.739904021 | 0.560770931 | 0.976259521 | 0.033184609 |
| SIRT1 | 0.172866452 | 0.065259476 | 0.457907603 | 0.000413217 |
| SIRT6 | 2.812020319 | 1.003427943 | 7.880444558 | 0.049244027 |
| SST | 1.232762412 | 1.00772707 | 1.508050353 | 0.041869699 |
| STAT5B | 0.175094643 | 0.065587174 | 0.467440998 | 0.000505386 |
| SUN1 | 0.345680149 | 0.152295992 | 0.78462187 | 0.011087284 |
| TCF3 | 2.916751026 | 1.114118551 | 7.636024497 | 0.029254649 |
| TFAP2A | 1.503189118 | 1.155643759 | 1.955254382 | 0.002379313 |
| TOP2A | 1.534441433 | 1.199882614 | 1.962284047 | 0.000644551 |
| TOP2B | 0.261256115 | 0.092947768 | 0.734334551 | 0.010909467 |
| UBE2I | 0.361061571 | 0.144610321 | 0.901494836 | 0.029102604 |
| UCHL1 | 1.297494968 | 1.008861868 | 1.668705345 | 0.042489834 |
| UCP2 | 1.801297506 | 1.064153554 | 3.049064389 | 0.02841478 |
| UCP3 | 0.6253345 | 0.393248468 | 0.994392271 | 0.047286397 |
| VEGFA | 2.380207459 | 1.499417909 | 3.778391278 | 0.000235043 |
| YWHAZ | 4.859360864 | 1.610543554 | 14.66175066 | 0.005019508 |
